# Characteristics and management of post‐circumcision Urethrocutaneous Fistula: a retrospective study in surgical units in Cameroon

**DOI:** 10.1002/bco2.391

**Published:** 2024-05-24

**Authors:** Landry Oriole Mbouche, Achille Aurèle Mbassi, Junior Barthelemy Mekeme Mekeme, Dorcas Nyanit Bob, Joseph Lionel Ndjock, Emmanuel Njuma Tamufor, Faustin Mouafo Tambo

**Affiliations:** ^1^ Department of Pediatric Surgery and subspecialties, Yaoundé Gyneco‐Obstetric and Pediatric Hospital University of Yaoundé I Yaoundé Cameroon; ^2^ Department of Urology, Yaoundé Central Hospital Higher Institute of Health Sciences Bangangté Cameroon; ^3^ Department of Urology, Yaoundé Central Hospital University of Yaoundé I Yaoundé Cameroon; ^4^ Department of Pediatric Surgery, Yaoundé Central Hospital University of Yaoundé I Yaoundé Cameroon

**Keywords:** Cameroon, characteristics, outcomes, post‐circumcision, surgical repair, urethrocutaneous fistula

## Abstract

**Background:**

Urethrocutaneous fistula (UCF) is one of the major complications of circumcision. The risk factors associated with UCF are not clear‐cut but its repair remains a challenge for urological surgeons. The aim of this study was to highlight the epidemiological, and clinical features and outcomes obtained from the management of UCF in the context of a country with limited medical resources where ritual circumcision is widely practiced.

**Patients and methods:**

From February 2010 to December 2022, 35 patients underwent surgical repair for post‐circumcision UCF in two tertiary hospitals in Yaounde, Cameroon. Simple closure, Thiersch‐Duplay‐Snodgrass and Mathieu techniques were performed.

**Results:**

The mean age of patients was 7.4 ± 4.1 years with a range of 2 to 21 years; the median age at circumcision was 24 months (12; 48). Most (95%) of circumcisions were performed by paramedical staff. The majority of patients (n = 26) consulted for a bifid stream, Three‐quarters of fistulae were located at the corona. Small fistulae represented 74.28% (n = 26) of cases as opposed to large fistulae (25.71%). More than 70% of patients underwent a simple closure. The therapeutic results were satisfactory in 91.4% of cases (n = 32) after an average follow‐up of 91.85 ± 51.92 months. There were no statistically significant differences between the patients with coronal fistula and patients with distal penile fistula concerning demographic, clinical and surgical characteristics.

**Conclusion:**

Urethrocutaneous fistula is a major and frequent complication of circumcision mostly practiced by non‐qualified personnel on children aged 24 months. The usual presentation is micturition with a bifid stream occurring on average 3 months after circumcision. Coronal fistulas are the commoner location. Simple closure, Thiersch‐Duplay‐Snodgrass and Mathieu technique appear to be safe with the advantages of low recurrence rate. An accurate diagnosis with a timeframe respecting the principles of fistula surgery combined with regular follow‐up is mandatory for good long‐term results with a low recurrence rate. Further prospective studies on the factors affecting the formation of urethrocutaneous fistula should be performed to prevent this complication of circumcision.

## 1 INTRODUCTION

In popular belief, circumcision is considered a minor or even trivial procedure. Male circumcision is performed at various ages by many potential providers in many areas of the world as part of cultural, religious and/or medical practices.^1^ There is a widespread lack of awareness regarding the complications of circumcision although it is the most common surgical procedure worldwide.^2^ Several authors have reported the occurrence of complications after circumcision.^1^, ^3^, ^4^ While some of these complications respond favourably to medical treatment or monitoring, others require additional surgery. Urethrocutaneous fistula (UCF) is one of the major complications of circumcision. This is an abnormal communication of the urethra with the skin, causing urine to leak through one or more abnormal orifices. The risk factors associated with UCF are incompletely clear‐cut, although it is commonly found following procedures performed by nurses in general, private hospitals and maternity homes.^5^ UCF repair remains a challenge for urological surgeons. The two most common surgical techniques currently used for UCF repair are simple closure and skin flap.^6^ We have experimented with three techniques in two tertiary hospitals considered to be reference centres in Yaoundé, Cameroon; the Yaounde Gynaeco‐Obstetric and Paediatric Hospital (YGOPH) and the Yaounde Central Hospital (YCH). Surgically cases using the simple closure, Thiersch‐Dupay‐SnodGrass and Mathieu technique were collated over a period of 12 years. The aim of this study was to highlight the clinical features and outcomes obtained from the management of UCF in the context of a country with limited medical resources where ritual circumcision is widely practiced.

## 2 PATIENTS AND METHODS

### 2.1 Study Population

This was a retrospective cross‐sectional study of 35 patients with UCF who underwent surgical repair in two tertiary hospitals of Yaoundé, Cameroon, between February 2010 and December 2022. The study was approved by the Ethics Committee of Yaounde Gynaeco‐Obstetric and Paediatric Hospital (Approval No.410/CIERSH/DM/2023, 30 March 2023) and Yaounde Central Hospital (Approval No. 2023/AR/MINSANTE/SG/DHCY/UAF, 13 February 2023). All patients who had surgery at the two reference centres were included. Those with a follow‐up time of less than 3 months and patients lost to follow‐up were excluded. The sample size was calculated using Fisher's unlimited population formula (N = z^2^ × p × [1‐p] /ε ^2^) where **Ζ** is the z‐score set at 1.96, which corresponds to a 95% confidence level, **ε** is the margin of error set at 5%, **p** is the prevalence of circumcision complications. According to WHO data we used a complication rate of 2%.^2^ The total sample size yielded 30 patients.

### 2.2 Surgical procedures and postoperative management

**FIGURE 1:**
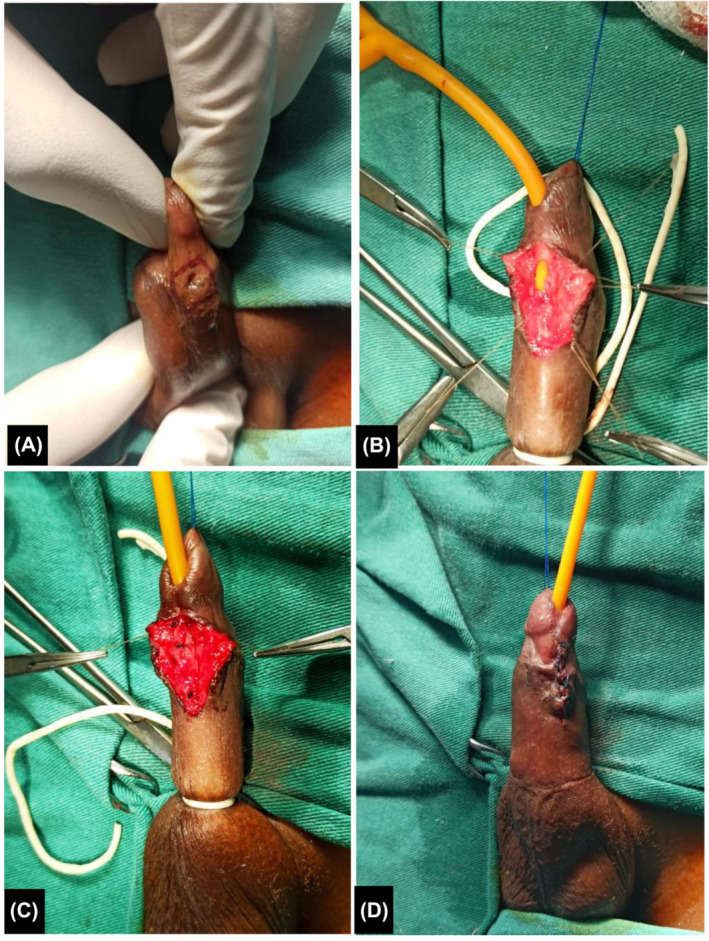
Intra‐operative view of simple closure procedure of a small UFC in a 6‐year‐old male. A. Perifistula incision line; B. Urethrocutaneous splitting (tourniquet at the root of the penis); C. Closure in two layers; D. Final appearance.

The children were initially seen on an outpatient basis. After a clinical assessment, the fistula was classified. A pre‐operative assessment and a pre‐anaesthetic evaluation were carried out before scheduling the operation. All operations were performed under general anaesthesia and aseptic conditions according to established surgical procedures. All the surgeries on our patients were performed by 3 senior surgeons. The choice of surgical technique depended on the size of the UCF, its location and the surgeon's preference. The fistulas were classified into 2 categories according to size: small fistulas (≤5 mm) and large fistulas (>5 mm). All fistulas were repaired at least one year after circumcision. Simple closure by urethrocutaneous splitting and coverage with dartos fascia was performed for small fistulas (figure 1). Large fistulas were repaired by the classic Thiersch‐Duplay technique along with Snodgrass' artifice (Thiersch‐Duplay‐Snodgrass) or the Mathieu skin flap procedure. Simple closure was performed for coronal UCF in both centres. While in YCH, Thiersch‐Duplay‐Snodgrass was the chosen technique for large‐size fistula, in YGOPH, Mathieu flap was preferred.

For the operative procedure, the patient was positioned supine. A transgranular stitch of prolene or ethilon 4/0 was made to expose the ventral surface of the penis. A tourniquet was placed at the root of the penis and a transurethral catheter was inserted.

For simple closure, a circular line was marked 5 mm around the fistulous orifice. A peri‐fistula incision was made with a scalpel and the skin was separated from the subcutaneous tissue after infiltration with 0.9% saline along the incision line to facilitate dissection. Once the healthy urethra is reached, it is sutured using simple interrupted 5/0 polydioxanone or polyglactin stitches. A covering layer using dartos was mobilized with scissors and folded over the first layer, staggering the suture line. The skin was closed with interrupted stitches using the same material. A semicompressive dressing was made using bacitracin ointment, vaseline gauze, sterile gauze and adhesive bandage. The transurethral catheter was folded back and fixed to the abdomen with an adhesive bandage.

With the Mathieu technique,^7^ we began by measuring the dimensions of the urethral defect. Upstream of this defect, a rectangular incision was made with the same dimensions. After infiltrating the skin with 0.9% saline using a 25G butterfly needle. The skin flap was removed by dissection with scissors and then folded forward over the urethral defect. The edges of the flap were then sutured to the edges of the native urethra using simple interrupted stitches of 5/0 polydioxanone. All the sutures were overlapped by the dartos or vaginal layer, depending on accessibility. The skin closure and dressing were both similar to the simple closure procedure.

Regarding the Thiersch‐Duplay‐Snodgrass technique also known as tubularized incised plate (TIP).^8^, ^9^ The procedure began by making two parallel incisions on either side of the fistulous orifice, then a longitudinal incision was made in the urethral bed using a scalpel to enlarge and deepen it. The two edges were closed around the transurethral catheter with interrupted stitches of 5/0 polydioxanone or polyglactin. The next stage of the procedure was spent on meatoplasty followed by repair of the glans penis. The covering layer, skin closure and dressing followed the same principles used for previous procedures.

After surgery, patients received antibiotic prophylaxis with Amoxicillin‐Clavulanic Acid. To control bladder spasms and erections, oxybutinin and diazepam were used up to catheter removal, respectively. On the fourth postoperative day, the surgical wound was uncovered and a thick film of bacitracin‐neomycin ointment was applied to it several times a day; sometimes beyond the removal of the urinary catheter. After catheter removal, the urine stream was appreciated. Follow‐up of patients after discharge from hospital was scheduled at 1 month, 3 months, 6 months, 1 year and then annually. On clinical examination, we were able to assess the urine stream, the surgical scar and the cosmetic appearance of the penis. The medical records of the enrolled patients were reviewed to evaluate data pertaining to demographic, clinical (age at circumcision, age at diagnosis, conditions for performing circumcision, urine stream, delay before consultation, location and size of the fistula), surgical treatment and outcome (delay before surgery, surgical procedure, hospital stay, urinary catheter duration and complications).

### 2.3 Statistical Analysis

Statistical analyses were conducted using Epi‐Info 7 software. The frequency and percentage of the categorical variables were presented. Continuous variables were presented as means ± standard deviations or medians with their interquartile range (25; 75 percentile) according to their normal or non‐normal distribution. Comparisons between the two groups were done using the Wilcoxon Mann–Whitney test for non‐parametric continuous variables and Student's t‐test for parametric quantitative variables, whilst qualitative data were compared using the chi‐squared test. The confidence interval (CI) was set to 95% and the accepted margin of error was set to 5%. The p‐value is considered statistically significant at p ≤ 0.05.

**FIGURE 2:**
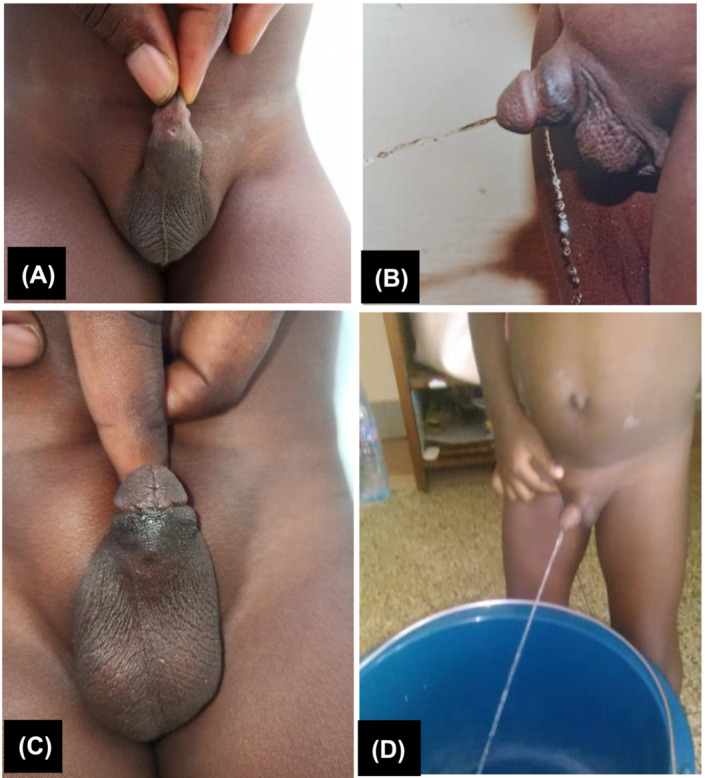
Presentation of UFC before and after treatment in a 6‐year‐old male. A. Small coronal UFC; B. Abnormal bifid stream at onset; *C. Fistula* closed on postoperative day 10; D. Single, normal micturition stream on postoperative day 10.

The efficacy of the surgical treatment was based on operative tolerance, length of hospital stay, occurrence of complications and quality of urine stream (figure 2). The success rate was defined as the proportion of patients operated on only one who did not require further surgery and who presented a satisfactory functional result.

## 3 RESULTS

**FIGURE 3:**
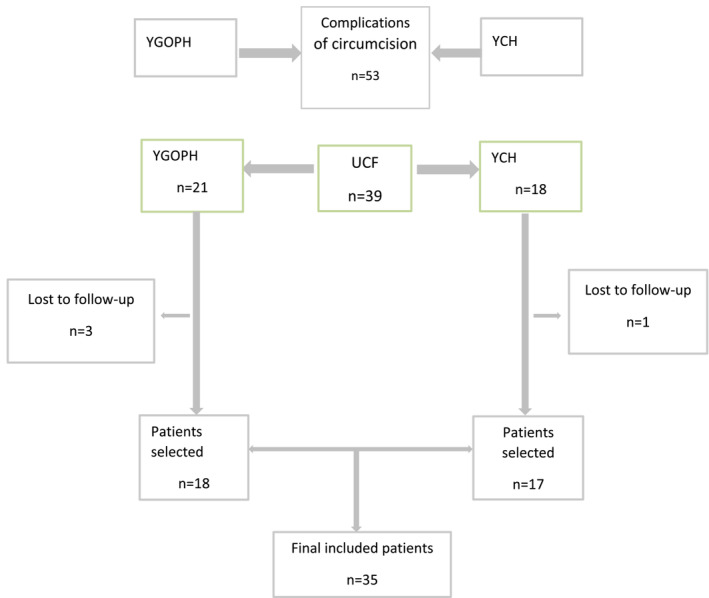
Flow chart of the patient selection.

During this study, we recorded 53 cases of complications following circumcision. Of these, 39 (73.58%) patients had a UCF. Fourteen cases of other complications including bleeding (7), glans amputation (2), tetanus (1) and meatal stenosis (4). We finally selected 35 cases including 18 at YGOPH and 17 at YCH (figure 3). There were four patients lost to follow‐up.

The mean age of patients at surgery was 7.4 ± 4.1 years with a range of 2 to 21 years. The median age at circumcision was 24 months (12; 48). The mean time between circumcision and the onset of symptoms was 3.68 ± 1.56 months and ranged from 1 to 6 months. Eight (30.76%) children from the centre region of Cameroon were circumcised before the age of 12 months and 18 (69.23%) other children were circumcised after the second year of life.

Among children from the western region, 3 (37.5%) had been circumcised before their first birthday and 5 (62.5%) after 24 months. The sole child from the north region was circumcised at the age of 5. Ninety‐five percent of circumcisions were performed by paramedical staff under local anaesthesia by using the forceps‐guided method. The majority of patients (n = 26) consulted for a bifid stream, part of which was through the normal route and the other on the ventral face of the penis. Nine (25.71%) patients had only a downward urine stream. Twenty‐six fistulae were located at the level of the corona and nine fistulae were in the distal penis. A 15 years old patient had a double corona fistula. Small fistulae represented 74.28% (n = 26) of cases and large fistulae comprised the remaining 25.71%.

Surgically, 25 (71.43%) patients underwent simple closure of the fistula, 7(20%) patients were operated on using the Thiersch‐Duplay‐Snodgrass technique and the Mathieu procedure was applied to 3 (8.57%) others. Urinary catheters were inserted in all patients, with a mean time to removal of 10.28 ± 1.17 days (10–15). The average hospital stay was 9.65 ± 1.43 days (3–10). The mean time from diagnosis to surgery was 3.88 ± 1.72 years (1–13). There were no statistically significant differences between the patients with coronal fistula and patients with distal penile fistula concerning demographic, clinical and surgical characteristics (Table 1).

**TABLE 1:** Patients' demographic, intraoperative and postoperative data.

Variables	Coronal UCF (n = 26)	Distal Penis UCF (n = 9)	p‐value
**Demographic**			
Age; years, mean (SD; range)	7.30 (4.38; 2–21)	7.55 (3.08; 4–12)	0.54^#^
Age at circumcision; months, median (IQ1; IQ3)	24(12;48)	36 (24;72)	0.44^#^
Circumcisers			
Healthcare professionals: 33 (95%)			
Non healthcare professionals: 2 (5%)			
Consultation time; months, mean (SD; range)	3.61 (1.44; 1–6)	3.88 (1.96; 1–6)	0.77^#^
Consultation time; months, median (IQ1; IQ3)	3 (3;5)	4 (2;6)	
Time to surgery; years, mean (SD; range)	3.92 (1.99;1–13)	3.77 (0.44; 3–4)	0.69^#^
Time for surgery; years, median (IQ1; IQ3)	4 (3;4)	4 (4;4)	
**Intraoperative data**			
Type of surgery			
Simple closure	25 (96.15%)	0	
Thiersch‐Duplay‐Snodgrass	0	7 (77.77%)	
Mathieu	1 (3.84%)	2 (22.22%)	
**Postoperative data**			
Catheterization time; days, mean (SD; range)	10.19 (0.98; 10–15)	10.55 (1.66; 10–15)	0.42^#^
Hospital stay; days, mean (SD; range)	9.53 (4.65; 3–10)	10.00 (0.00;10)	0.39^#^
Complications			
Total	3 (8.5%)	0	
Recurrence	2 (5.7%)	0	
Urethral stricture	1 (2.8%)	0	
Simple closure	3/25	0	
Thiersch‐Duplay‐Snodgrass	0/7	0	
Mathieu	0/3	0	
Follow‐up duration	91.85 ± 51.92 months (5.35–251.47)		

(UCF, Urethrocutaneous fistula; SD, Standard deviation; IQ, Interquartile; ^**#**^T‐test).

The therapeutic results were satisfactory in 91.4% of cases (n = 32) after an average follow‐up of 91.85 ± 51.92 months (5.35–251.47). We recorded 3 (8.57%) cases of complications classified as Clavien‐Dindo grade IIIb. There were two recurrences and one urethral stricture; all from coronal UCF. The two recurrences underwent simple closure and the urethral stricture was repaired in two stages.

## 4 DISCUSSION

**FIGURE 4:**
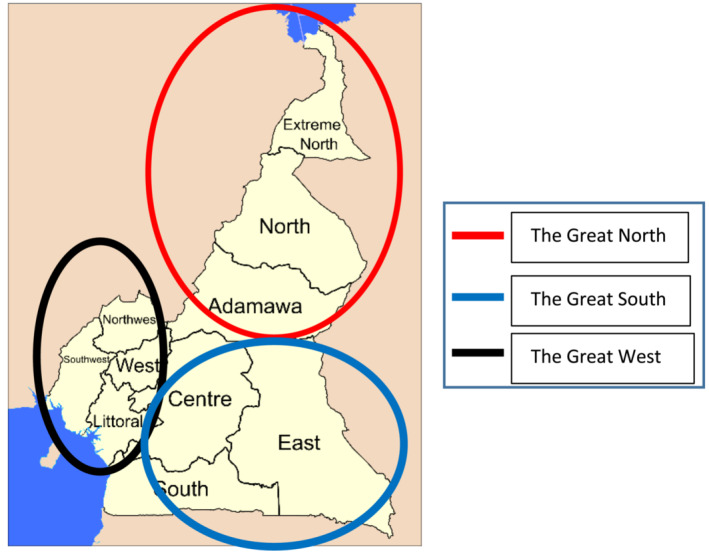
Map of Cameroon showing 10 administrative regions divided into 3 cultural parts. *Source: adapted from Wikipedia Free Encyclopedia (* http://en.wikipedia.org/wiki/Image *:Provinces_of_Cameroon_EN_svg).*

Complications related to circumcision vary from one study to another, reaching up to 21%.^10^, ^11^ There is controversy about the complication rates of neonatal and early childhood circumcision; while some authors report a high rate of circumcision complications in newborns,^10^, ^12^ other studies have shown an increased frequency of circumcision complications in children.^13^, ^14^ The prevalence of post‐circumcision complications in some African studies shows an upward trend, as reported by Okeke^15^ and Osuigwe^16^ in Nigeria with 20.2% and 24.1%, respectively. However, Manji^17^ and Auvert^18^ in Tanzania and South Africa respectively recorded lower complication rates of 2.8% and 3.8%. The choice of circumcision method depends on the physician's level of comfort and training. Each instrument and technique carries its own benefits and complication risks.^4^ The qualifications of the personnel performing the circumcision have an impact on the occurrence of complications. A high complication rate is mostly found when paramedical staff carry out the circumcision.^15^, ^16^ However, there are fewer complications in the hands of trained staff.^17^, ^18^ Most of the circumcisions in our setting are performed by nurses but it's difficult to meet the practitioner who often hides their identity to avoid serious medico‐legal problems after surgery, leaving parents or guardians to come to specialized centres without any information about the procedure. There is no standard age for circumcision in Cameroon. It is mainly practiced by paramedical staff^3^ and depends on the cultural considerations specific to each region. These regions are divided into three parts (the Great North, the Great South and the Great West) (Figure 4). While in the Great North, it is performed on older children and adolescents during initiation rites; in the Great West, circumcision is usually performed on newborns; in the Great South, it is performed after the neonatal period on infants. However, our customs are increasingly influenced by the environment in which we live. Although the complication rate associated with this procedure remains high in our setting, circumcision is performed both in hospital and at home by people who are sometimes unqualified. In fact, parents generally remain silent when asked about the conditions under which their children underwent circumcision. This unsupervized activity of the most common surgical procedure in the world is the harbinger of most of the complications observed. In our series, urethrocutaneous fistula was the main complication of circumcisions, accounting for 39 patients out of 53 (73.58%). In 2016, another study carried out in Yaounde, Cameroon on 15 cases showed an annual incidence of circumcision complications of 2.5 cases per year.^3^ In that study urethral fistula was predominant, involving 53%of cases. Osifo and Takure in Nigeria also reported a frequency of post‐circumcision urethrocutaneous fistulas that reached 21.1% and 74% of their cases, in Benin city and Ibadan, respectively.^11^, ^19^

The complications of circumcision are more often minor than major^10^; this is the case for UCF, which most of the time requires surgical repair. Although none of the cases in our series were premature, but had their circumcision during their first birthday, UCF is the result of damaging the urethral wall by cutting, crushing, or suturing. Moreover, thinner tissue overlying the urethra in immature genitalia may predispose boys to injury. This situation is often attributed to untrained personnel with poor surgical technique.^20^ The mechanism of occurrence of UCF is not fully elucidated but some theories and explanations are widely described.^6^, ^11^, ^20^, ^21^ The penile urethra is superficial, separated from the skin only by 5 to 6 mm of spongy tissue, especially at the level of the frenular artery^22^ which is the most common location for fistulas. The arterial supply of the distal part of the urethra arises from branches of the internal pudendal artery. As a result, the urethra has a borrowed vascularization system. If this blood supply is compromised, ischaemia, tissue necrosis and breakdown would result in fistula formation. The particular feature of this analysis is that all the small fistulas are coronary fistulas, whereas the larger fistulas are mainly distal penile fistulas. In cases of distal penile fistula, large amount of ischaemia tissue and infection of the wound could be associated trigger factors.^20^ This is due to a dirty environment, which includes non‐sterile gloves wearing, instruments that are poorly or non‐sterilized. It is therefore essential that follow‐up visits should occur within seven days of surgery to assess the progress of healing and to look for signs of infection.^2^ Probably, because of these ischaemic lesions, we found that the time elapsed between circumcision and the appearance of the fistula goes beyond 30 days. These findings are consistent with other studies in which the time taken to diagnose UCF was equally long, ranging from 14 days to 108 months for Ukwu in Nigeria and between 14 and 27 days for Lucas in 15 other African countries.^5^, ^20^ A case of a patient treated at the age of 18 years has also been reported.^21^ This situation of late diagnosis is thought to be partly due to ignorance of the situation by parents or guardians, who do not always observe the micturition of infants because they are constantly wearing diapers.^5^ On the other hand, complications that require emergency treatment will reach the hospital sooner, whereas others such as UCF, which are not life‐threatening, may lead to late consultation; and even when the diagnosis has been made, surgery may be performed a long time later. Furthermore, the likelihood of spontaneous closure of the fistula can keep the parents waiting longer, and sometimes even make them forget or be laxer about returning to the hospital.

The classic manifestation reported in the literature is a bifid ventral urine stream.^1^, ^3^ However, surgical exploration may reveal more than one fistulous opening as in the case of one of our patients who had a double fistula orifice. Sancaktutar^21^ in Turkey described an 18 years old man with four fistula orifices around the corona of the penis. Although rare with an unknown pathological mechanism, both cases of multiple urethrocutaneous fistula have been observed in adolescents. This may be due to the long‐standing nature of the fistula in these patients. The coronal location is predominant over the distal penile position for fistulas, as previous studies have already reported.^11^, ^19^, ^23^ We also noticed that distal penile fistulas were of interest to infants over 36 months, whereas coronary fistulas involved infants under 24 months. Perhaps this is due to a poorly handled technique and an environment unsuited to an optimal surgical practice.

Concerning surgical repair of UCF, some surgeons prefer a direct closure whereas others surgeons prefer skin flaps.^6^ Among the various techniques, simple closure, the Tiersch‐Duplay‐Snodgrass technique and the Mathieu flap are widely described by experts.^5^, ^24^, ^25^, ^26^ Indeed, the choice of fistula repair technique depends on the size of the fistula, the condition of the penile skin, the degree of scarring and the surgeon's preference.^6^, ^19^, ^20^, ^26^ Our surgical indications are in accordance with those of Ceylan et al in Turkey, who also insisted on the age of the patients, admission time, and the condition of the local penile skin as their selection criteria.^26^ This is not the approach taken by Baskin.^24^ In his series of eight patients with UCF located on the distal penile shaft or at the coronal margin, he showed that UCF can be successfully repaired without recurrence by splitting the glans and forming a neourethra from a vascularized pedicle flap of the penile skin.

According to the literature, simple closure of a UCF is technically easy, not time‐consuming and does not compromise the original shape of the penis.^27^, ^28^ However, the success rate varies according to series. It is shown in studies reported by Takure, Ikuerowo and Diallo with success rates reaching 84% (n = 20), 100% (n = 9), and 78.9% (n = 30), respectively, indicated for small‐size coronal post‐circumcision UCF.^19^, ^25^ Applied to repair of post‐hypospadias urethrocutaneous coronal fistula, simple closure technique gives results comparable with those of post circumcision UCF. Thus Dekalo and Srivastava have reported a successful rate of 83% (n = 81) and 77% (n = 13), respectively.^29^, ^30^ Our series recorded a successful rate of 88% (n = 25) for simple closure. This means that this procedure can have a significant failure rate of up to 20% and beyond. It is also an important point to consider that the scar tissue left by circumcision can compromise the success of this reconstructive surgery. Therefore, the key factors for success in this type of surgery are gentle tissue handling, the use of fine sutures, a subepithelial urethral closure and the interposition of tissue to avoid direct contact with suture lines.^31^

One of the major difficulties in repairing large UCFs is skin coverage. When the underlying tissue is used to close the fistula, a skin gap is created. This gap is usually managed either by a longitudinal penile skin incision used as a relaxing incision to reduce the skin tension of the closed fistula area^28^ or by mobilizing skin flaps. On the other hand, some surgeons prefer other approaches using flaps, such as the Mathieu technique and tubularized incised plate (TIP) urethroplasty that we successfully performed on patients with large‐size UCF without complications. Whilst there are few studies on the surgical treatment of post‐circumcision UCF using these two techniques, complication rates have been reported to be 2–13% for both procedures following hypospadias repair.^32^ The most commonly reported serious complications after hypospadias surgery are urethral stricture and UCF.^32^

In response to the question of when to operate on a post‐circumcision UCF, the majority of studies agree on a minimum delay of 6 months.^24^, ^26^, ^27^ This is the timeframe to allow resolution of tissue oedema and scar maturation.^17^ In our study, the time taken for surgery exceeded 6 months firstly to promote spontaneous closure of the fistula and secondarily to ensure that inflammatory processes subside and provide a soft peri‐fistula tissue for reconstruction. Urinary diversion, the use and duration of the urethral catheter remain a subject of debate.^6^, ^26^ In a recent study, Kumar et al.^33^ showed that the occurrence of long‐term complications of TIP hypospadias repair was not affected by the early removal of the bladder catheter. In our series, the transurethral catheter allows us to divert urine away from the reconstructed area and thereby avoid provoking an inflammatory reaction that could weaken it, with the risk of recurrence. In this respect, the patients are hospitalized until the removal of the urinary catheter to monitor the quality of local care and the evolution of the surgical wound, which is provided in a hospital environment, as opposed to an unhealthy community environment which is very often ill‐suited to optimal post‐operative care. Incidentally, the objective of this reconstructive surgery is to have a satisfactory cosmetic and functional of the penis. Indeed, recurrence after UCF repair can occur regardless of the technique used. Rarely, post‐operative complications can occur following trauma but, we recorded a case of urethral stricture probably due to the traumatic removal of urethral catheter with the balloon insufficiently deflated by a resident. This incident may cause a urethral injury with resultant stricture healing.

The debate about who is qualified to perform circumcisions is still ongoing in Cameroon. During the Cameroon National Medical Conference held in December 2022,^34^ discussions on the subject led to the conclusion that there were not enough doctors authorized to perform circumcisions throughout the country. As a result, paramedical healthcare staff need to be trained as much as possible to carry out circumcisions without complication; similarly, simulation workshops using models have been set up, enabling medical students to become familiar with the techniques. The procedure, which has long been practised and routinely performed by unqualified healthcare professionals and non‐healthcare professionals, now seems to be taking a different course, as it is regularly included in surgical operatory schedules in Cameroon.

Our study is subject to certain limitations due to its retrospective nature, such as the absence of information on the circumcision procedure and the conditions that led to the fistula. Circumcision is still widely practised by unqualified healthcare and non‐healthcare professionals, and even clandestinely. This situation makes it difficult to assess a clear trend in the practice of circumcision and its complications.

## 5 CONCLUSION

Despite a widespread practice in the world with satisfactory overall results, circumcision can give rise to severe complications, such as UCF. In our setting, it is a major and frequent complication of circumcision mostly practiced by non‐qualified personnel on children aged 24 months. The usual presentation is a double stream urine occurring on average 3 months after surgery. Coronal fistulas are the commoner location. Simple closure, Thiersch‐Duplay‐Snodgrass and Mathieu technique appear to be safe with the advantages of low recurrence rates. An accurate diagnosis with a timeframe respecting the principles of fistula surgery combined with regular follow‐up is mandatory for a better long‐term result with a low recurrence rate. Further prospective studies on the factors affecting the formation of UCF should be performed to prevent this complication of circumcision.

## AUTHOR CONTRIBUTIONS

MLO, MAA were major contributor to the conception and design of the study and in drafting the manuscript. MLO, MMJB, NBD, NJL and NTE analyzed and interpreted the patient data and MTF provided administrative and technical support. All of the authors read and approved the final manuscript.

## CONFLICT OF INTEREST STATEMENT

The authors declare that they have no competing interests.

## FUNDING

None.

## AVAILABILITY OF DATA AND MATERIALS

The dataset analysed in this study is available from the corresponding author by request.

## ETHICS DECLARATIONS

## ETHICS APPROVAL AND CONSENT TO PARTICIPATE

This study was approved by the ethics committee of Yaounde Gyneco‐Obstetric and Pediatric Hospital (reference no.410/CIERSH/DM/2023) and Yaoundé Central Hospital (reference no.2023/066/AR/MINSANTE/SG/DHCY/UAF). Informed consent was obtained before surgery from the parents of all minor participants (less than 16 years of age) and from participants (above age of 16) for inclusion in the study. This research has been carried out per current Cameroonian ethical standards and The Code of Ethics of the World Medical Association. All methods were carried out following the guidelines of the Declaration of Helsinki.

## CONSENT FOR PUBLICATION

Not applicable.
